# Using Teleost Fish to Discern Developmental Signatures of Evolutionary Adaptation From Phenotypic Plasticity in Brain Structure

**DOI:** 10.3389/fnana.2020.00010

**Published:** 2020-03-18

**Authors:** Zachary J. Hall, Vincent Tropepe

**Affiliations:** ^1^Department of Biological Sciences, University of Alberta, Edmonton, AB, Canada; ^2^Department of Cell and Systems Biology, University of Toronto, Toronto, ON, Canada

**Keywords:** brain, evolution, plasticity, teleost, neurogenesis

## Abstract

Traditionally, the impact of evolution on the central nervous system has been studied by comparing the sizes of brain regions between species. However, more recent work has demonstrated that environmental factors, such as sensory experience, modulate brain region sizes intraspecifically, clouding the distinction between evolutionary and environmental sources of neuroanatomical variation in a sampled brain. Here, we review how teleost fish have played a central role in shaping this traditional understanding of brain structure evolution between species as well as the capacity for the environment to shape brain structure similarly within a species. By demonstrating that variation measured by brain region size varies similarly both inter- and intraspecifically, work on teleosts highlights the depth of the problem of studying brain evolution using neuroanatomy alone: even neurogenesis, the primary mechanism through which brain regions are thought to change size between species, also mediates experience-dependent changes within species. Here, we argue that teleost models also offer a solution to this overreliance on neuroanatomy in the study of brain evolution. With the advent of work on teleosts demonstrating interspecific evolutionary signatures in embryonic gene expression and the growing understanding of developmental neurogenesis as a multi-stepped process that may be differentially regulated between species, we argue that the tools are now in place to reframe how we compare brains between species. Future research can now transcend neuroanatomy to leverage the experimental utility of teleost fishes in order to gain deeper neurobiological insight to help us discern developmental signatures of evolutionary adaptation from phenotypic plasticity.

## Evolutionary Adaption vs. Phenotypic Plasticity in Neuroanatomy

Studies comparing the brains of different species enable us to elucidate the extent to which evolutionary forces shape brain structure and, by extension, the perceptual, cognitive, and behavioral functions the brain supports. Traditionally, brain evolution has been studied neuroanatomically, focusing on interspecific variation in either whole brain size or the size of individual brain structures. When comparing the brains of two or more species in this fashion, we assume that similarities in neuroanatomy are a product of conserved neurodevelopmental processes across species and that differences arise as a product of evolution.

Conversely, studies comparing the brains of individuals of the same species enable us to elucidate the extent to which environmental forces shape brain structure and function. Such phenotypic plasticity is often studied neuroanatomically, in which changes in brain structure are associated with environmental factors such as nutrient availability and sensory experience along with genetic factors. When comparing the brains of two or more individuals in this fashion, we assume that similarities in neuroanatomy are a product of conserved neurodevelopmental processes within a species and that differences arise as a product of a rearing environment.

But what happens when neuroanatomical variation manifests similarly between and within species? If, for example, the olfactory bulb is enlarged in one species compared to another, is this enlargement a product of evolutionary forces or a product of differences in olfactory experiences between species during neurodevelopment? Without understanding the extremes of phenotypic plasticity in neuroanatomy within a species, can we affirm that observed interspecific variation in brain structure is primarily a product of evolution and not the different environments experienced by the individuals sampled representing each species? In this perspective article, we discuss the utility of teleost fish species as animal models in addressing evolutionary and environmental sources of neuroanatomical variation. First, we review past literature, focusing on important insights derived from the study of teleost comparative neuroanatomy, and more recently, neurodevelopmental plasticity in response to environmental factors within a species. We then highlight the difficulty in isolating the influence of evolution from the environment by studying only brain size, discussing neurogenesis as a common developmental mechanism that appears to underlie both inter- and intraspecific changes in neuroanatomy. Finally, we consider recent advances in identifying uniquely evolutionary sources of neuroanatomical variation isolated from phenotypic plasticity and propose additional future research directions that may help to further distinguish evolutionary and developmental forces shaping the brain.

## Teleosts as Model Species in Comparative Neuroanatomy

In general, average brain size has increased during vertebrate evolution and, while much of this is due to changes in overall body size, some of this variation is due to evolutionary forces acting on brain development beyond allometric constraints (Striedter, 2005). Such comparative neuroanatomy has generated hypotheses positing that evolutionary forces promoting larger brains both preceded and were necessary for the subsequent evolution of complex cognitive processes including social learning (van Schaik and Burkart, 2011) and tool manufacture (Emery and Clayton, 2005). However, focusing on variation in whole brain size has been criticized for concealing changes that may be occurring within the brain’s major constituent parts independently (Healy and Rowe, 2007). Accordingly, models of brain evolution have also considered how the sizes of distinct brain structures scale with overall brain size based on correlations with ontogeny, habitat complexity, and behavioral specialization (Finlay and Darlington, 1995; Barton and Harvey, 2000). We now appreciate that both mosaic evolution (evolution of brain regions relatively independent from one another) and developmental constraints (concerted evolution of brain regions) play fundamental roles in explaining the neuroanatomical variation that we observe in nature (Striedter, 2005).

The conceptual basis for models of brain evolution is built on a foundation of mostly descriptive neuroanatomical approaches (Northcutt, 2002). Due to their extensive phenotypic, behavioral, and ecological diversity (Streelman and Danley, 2003; Shumway, 2008), teleost fish have been used historically in studies associating brain structure to evolutionary adaptation. For instance, Huber et al. (1997) generated an extensive database of brain region morphology for 189 African cichlid species across three inland lakes. Using comparative analysis, they reported that species evolved to engage in agile prey capture behavior exhibit larger cerebella and optic tecta, a midbrain structure and primary recipient of retinal input, compared to species evolved to feed on relatively stationary mollusks and plants. The authors interpret this neuroanatomical difference as a product of evolutionary specialization to improve visual and motor capabilities in order to track and chase moving prey.

Complementary to this work, Kotrschal and Palzenberger (1992) found that bottom-feeding benthivore cyprinid species exhibit an evolutionary increase in the size of brain structures involved in processing chemosensory and olfactory input, consistent with relaxed evolutionary pressure on visual capabilities and increased importance for smell and taste while feeding along turbid lake bottoms. These investigations set the stage for the concept of brain ecotypes, in which brain morphology is specialized to improve sensory processing in the modality most critical for feeding success (Sylvester et al., 2010). Since this work, additional teleost studies have identified evolutionary patterns in brain morphology associated with habitat complexity, social organization (Pollen et al., 2007), sexual selection, and parental care across species (Gonzalez-Voyer and Kolm, 2010). Whereas almost all comparative studies in teleost neuroanatomy include whole brain size in these analyses, they also include additional analysis of brain components, and typically explain whole brain size findings as a product of changes in specific brain structures. For example, monoparental female care in cichlids is associated with the evolution of a larger whole brain (Gonzalez-Voyer and Kolm, 2010). However, brain component analysis reveals that, whereas most brain components studied are larger in these species, cerebellar and hypothalamic volumes decrease. Collectively, teleost fishes have been shown to be powerful models in comparative neuroanatomy, identifying patterns in both concerted and mosaic brain evolution associated with evolutionary forces across species. Common to all of these studies is the assumption that evolutionary pressure to improve certain types of sensory processing will drive changes in brain structure size and that such neuroanatomical measurements accurately capture species differences in the brain. However, research on phenotypic plasticity in teleost brains challenges this assumption, demonstrating that the environment similarly impacts brain structure within a species.

## Teleosts as Model Species in Studying Phenotypic Plasticity in Neuroanatomy

A common criticism of comparative approaches to neuroanatomy is that single species must often be represented by individual measurements collected from few brains. For example, the work of Huber et al. (1997) discussed above included 189 cichlid species represented by 216 brains, indicating that most species data was derived from a single, adult brain from a museum collection. By reducing species to single measurements we overlook potential intraspecific variation in brain structure. This oversight can either over- or underrepresent the inferred evolutionary contributions to differences in neuroanatomy between species. Because comparative work in teleosts focuses on the size of individual brain structures, is there evidence of phenotypic plasticity in these same measurements within a species?

Some of the first evidence demonstrating the capacity of the environment to shape the teleost brain structure came from comparisons between wild-caught and lab-reared fish populations. Salmon reared in a hatchery exhibit reduced olfactory bulb and telencephalon size compared to age-matched wild salmon from the same genetic cohort (Kihslinger et al., 2006). The first generation of female guppies reared in the laboratory from wild parents exhibited reduced telencephala and optic tecta compared to wild-caught fish (Burns et al., 2009). Because the laboratory environment is traditionally considered to lack much of the sensory stimuli animals would encounter in the wild, these findings generated hypotheses suggesting that neuroanatomical development in fish is influenced by sensorimotor experiences, particularly those with ethological value to the species studied (Gonda et al., 2011). Consistent with these hypotheses, both male guppies collected from regions of high predation and laboratory-reared male guppies exposed to olfactory and visual predator cues during development have larger brains as adults compared to unexposed males (Reddon et al., 2018). Thus, phenotypic plasticity may be an important factor in explaining neuroanatomical variation when comparing brains.

Another approach to studying intraspecific variation in teleost neuroanatomy has been to compare fish populations of the same species inhabiting different environments. These studies have revealed habitat-dependent correlations with brain size similar to studies both comparing lab- and wild-bred populations and correlating habitat and brain structure across species comparatively. For example, whole-brain size is larger in sunfish that occupy a littoral shoreline habitat vs. those that live in a pelagic habitat (Axelrod et al., 2018), and marine populations of nine-spined sticklebacks had larger olfactory bulbs and telencephala relative to pond populations (Gonda et al., 2009). A limitation to comparing populations in this manner is that genetic differences among different populations of the same species can translate into different brain morphologies (Ishikawa et al., 1999), complicating the assertion that environmental factors alone explain these differences in the brain. However, we believe it is critical to note that intraspecific variation in neuroanatomy identified both between populations and also in lab- and wild-reared individuals is similar in form and even, in some cases, magnitude to those described between species in comparative teleost work.

Perhaps, then, size alone is insufficient to separate inter- and intraspecific variation in brain structure. Instead, one might ask whether differences in brain structure between and within species are achieved *via* the same developmental mechanisms. Mechanistic work examining evolution and phenotypic plasticity in the teleost brain highlights the depth of the problem in relying on mature neuroanatomy alone in comparative work: neurogenesis, the production, and incorporation of new brain cells appears to be a common mechanism generating variation in neuroanatomy both between species, *via* evolutionary changes in neurogenic brain development, and within species, as a form of sensory experience-dependent neuroplasticity.

## Neurogenesis as a Common Mechanism of Evolutionary and Environmental Variation in Brain Structure

How do teleost brain regions get bigger in some species? In the vertebrates, the most discussed model of the evolutionary growth of brain structures is the “late equals large” model (Finlay et al., 2001). This model argues that vertebrate brains grow following a similar developmental sequence in which new neurons are generated and incorporated into different brain structures at different times. For a particular brain region to grow larger, the period of neurogenesis in which new neurons are added to that structure is protracted. This model has been used most popularly to explain the expansion of the cerebral cortex in humans (McKinney, 2002; Finlay and Brodsky, 2006). Whereas this model has been criticized for simplifying patterns of brain structure evolution (Barton and Harvey, 2000), opposing models similarly argue that regulating the timing and length of neurogenic periods underlies evolutionary changes in brain structure (reviewed in Montgomery et al., 2016). In teleosts, changes in the timing of neuron production within distinct brain regions are also thought to be the primary means through which differential brain growth between species occurs (Sylvester et al., 2011). If neurogenesis is the primary mechanism through which brain regions change size between species, then what role does neurogenesis play, if any, in influencing brain structure within species?

Traditionally, the influence of the environment and specific sensory experience on brain development has been assumed to manifest as synaptic plasticity in pre-existing neurons (Knudsen, 2004; Hensch, 2005). In part, this belief appears to stem from the assumption that, at least in mammals, neurogenesis is an embryonic process that is largely complete by birth, before an animal is exposed to the external environment. However, since the discovery of adult neurogenesis in the mammalian brain (Altman and Das, 1965), a growing body of work demonstrates that neurogenic processes continue to shape the brain well beyond embryogenesis (Feliciano et al., 2015). Furthermore, teleosts exhibit extensive neurogenesis in the brain throughout life compared to mammals (Zupanc and Horschke, 1995; Lindsey and Tropepe, 2006), indicating that neurogenesis may be a life-long neurobiological substrate for brain growth in fish.

One of the first studies to document experience-dependent modulation of developmental neurogenesis compared the telencephala of salmon reared in environments differing in water flow velocity and physical structure (Lema et al., 2005). The authors reported that the rearing environment affected neural progenitor cell proliferation rates in the telencephalon throughout development. Whereas the authors did not find a difference in telencephalon size associated with these changes in neurogenesis, this may be due to their environmental manipulation which enriched water flow in one condition and physical environment in another, preventing conclusions on the importance of a single type of sensory experience on telencephalon development.

Since this finding, work on adult zebrafish has identified visual, olfactory, and social experience-dependent modulation of neurogenesis (Lindsey and Tropepe, 2014; Lindsey et al., 2014). A limitation to these results in the context of our discussion here, however, is that analyses included only adult fish, which exhibit the lowest rates of neurogenesis compared to earlier in life. Thus, adult changes in neurogenesis would likely not translate into changes in brain size. Encouraged by these results, we tested whether similar sensory experience-dependent neurogenic modulation would occur postembryonically in zebrafish when neurogenesis persists at a much higher rate and is the primary driving force promoting brain growth (Cerveny et al., 2012; Furlan et al., 2017). We found that visual and sensorimotor experience regulated the neurogenic growth of the optic tectum and telencephalon in zebrafish, respectively (Hall and Tropepe, 2018a, b). Specifically, we found that rearing zebrafish larvae in a low-intensity light reduced the number of newly generated neurons that incorporate into the optic tectum. Anatomically, this reduced neuronal incorporation in the tectum reduced the size of the tectal neuropil, which is in part innervated by apical projections from the new neurons tracked in this study, in as few as 10 days of development (Hall and Tropepe, 2018a). In our second study, we found that restricting a zebrafish larva’s movement significantly reduced the proliferation of neural precursors in the dorsal telencephalon, reducing the size of the telencephalon in as few as 6 days (Hall and Tropepe, 2018b). Our work demonstrates not only the capacity for sensory experience to modulate neuroanatomy *via* neurogenesis, but also shows that experience shapes tectal and telencephalic anatomy intraspecifically, two brain regions reported exhibiting evolutionary specialization in size across teleost species (van Staaden et al., 1994; Huber et al., 1997).

Collectively, the work above identifies a critical issue with discerning evolutionary and environmental sources of brain size variation: variation in brain structure appears to manifest similarly between and within species at the level of both changes in brain size and the neurogenic developmental processes preceding them.

## Deciphering Phylogenetic Adaptation vs. Phenotypic Plasticity

The preceding discussion highlights the utility of teleost models for studying how evolutionary and environmental forces shape the brain. By limiting analyses to brain region size and rates of neurogenesis, however, we have been unable to discern the contributions of evolution and environment to neuroanatomy. One solution to revealing evolutionary signatures in the teleost brain has been to look even earlier in development, prior to changes in brain morphology and earlier developmental neurogenesis.

Research on mapping gene expression along the anterior-posterior and dorsal-ventral brain axes in related cichlid species has revealed species-specific differences in the timing of expression of these genes that subsequently developed into species differences in brain morphology (Sylvester et al., 2010). Cichlid species that develop brains supporting greater visual capacities for agile prey capture exhibit embryonic gene expression patterns that led to the relative growth of brain structures involved in processing vision, including the thalamus and optic tectum. Conversely, cichlid species developing brains supporting greater olfactory capabilities for benthic feeding exhibit embryonic gene expression patterns that led to the relative growth of ventral and anterior brain regions, including the subpallium and olfactory bulb. By interfering with the expression patterns of one of these genes, *Wnt*, using doses of lithium chloride, Sylvester et al. (2010) perturbed embryonic gene expression in such a way that an “olfactory-based” cichlid species would develop a brain anatomically reminiscent of a “visual-based” cichlid (Sylvester et al., 2011). This and other work showing how opposing *Hedgehog* and *Wingless* signaling pathways can regulate species difference in the structure of the fish telencephalon (Sylvester et al., 2013) have provided a new perspective in comparative neuroanatomy, identifying novel mechanisms through which evolution has shaped brain development manifesting as gene expression regulation in embryonic development, prior to (or coincident with) the onset of neurogenesis, changes in brain region size, and the processing of sensory experience.

We believe the next useful step is to connect these embryonic patterns of gene expression to prior work identifying species differences in mature neuroanatomy by refocusing on intermediary neurogenic developmental processes. Unlike prior work, however, we believe studying interspecific differences in developmental neurogenesis must recognize that neurogenesis itself is a multi-stepped process involving neural stem cells producing intermediate progenitor cells producing neurons, which may or may not survive long enough to integrate into neural circuitry (Lindsey et al., 2018). Accordingly, when a brain region is larger in an individual or species, this can be achieved through a multitude of different neurogenic mechanisms, such as changes in cell proliferation, fate, and survival, which in turn may generate mature brain structures with vastly different neuronal compositions. For example, we found that visual experience modulates optic tectum size by affecting the survival of all tested neuronal phenotypes equally (Hall and Tropepe, 2018a), suggesting that tectal size is proportionally scaled by visual experience. Does a similar proportional scaling occur across teleost species or do the tecta of different teleost species contain different proportions of neurons serving different functions?

Characterizing developmental neurogenesis in teleosts has revealed that different brain regions incorporate new neurons generated by distinct neural stem cell populations. For example, the dorsal telencephalon incorporates new neurons generated by radial glial neural stem cells whereas the ventral telencephalon primarily incorporates new neurons generated by neuroepithelial stem cells (Wullimann, 2009; Lindsey et al., 2018). We found that, at least intraspecifically, these neural stem cell niches can be regulated independently of one another (Hall and Tropepe, 2018a, b) and persist into adulthood (Lindsey et al., 2012). Such neuroanatomical modularity in developmental neurogenesis may be a novel avenue in which interspecific differences in brain structure may manifest through proposed models of mosaic brain evolution discussed above.

In a recent display of the power in connecting embryonic gene expression with subsequent changes in neurogenesis, Cárdenas et al. (2018) found that interspecific differences in embryonic *Robo* gene expression influence whether or not intermediate progenitor cells either abstain from dividing and differentiate into neurons or first divide before differentiating, ultimately doubling the number of neurons produced by a neural stem cell population. The authors suggest that such *Robo*-dependent increases in progenitor proliferation underlie the extreme forebrain expansion necessary to evolve a cerebral cortex in mammals (Kriegstein et al., 2006) compared to other vertebrates. Whereas analogous forebrain progenitors in zebrafish are assumed not to divide and instead strictly differentiate into neurons (Furlan et al., 2017), whether these neural progenitors exhibit differences in proliferative behavior across fish species during development as a mechanism underlying evolutionary specialization in telencephalon size may be a promising future research avenue.

## Conclusion

With the advent of comparative neurobiological work at the levels of both embryonic gene expression and mature neuroanatomy and the growing understanding of developmental neurogenic processes in teleosts, we are poised to weave molecular embryonic, developmental, and neuroanatomical techniques to modernize our understanding of vertebrate brain evolution. By adopting integrative approaches through which early patterns of gene expression are translated into neurogenic growth processes in development that ultimately culminate in mature brain structures, we will develop a novel understanding of how evolutionary signatures in species-specific embryonic gene expression develop into evolutionary signatures in brain structure and function. As discussed above, teleosts have made fundamental contributions towards unveiling the problem of isolating evolutionary and environmental contributions in the brain by relying on neuroanatomy alone; however, teleosts have also provided some of the only insight illuminating a path towards new integrative approaches to overcome this problem. Teleosts remain one of the most, if not the most, accessible clades to collect multiple species and study their development in the lab or in the wild, enabling a complete gene-to-development-to-neuroanatomy approach across species and leading the way for a new understanding of evolved differences in the central nervous system.

## Author Contributions

ZH and VT conceived and wrote the manuscript.

## Conflict of Interest

The authors declare that the research was conducted in the absence of any commercial or financial relationships that could be construed as a potential conflict of interest.
